# Editorial: Advances in food processing and analysis: product quality and green revolution

**DOI:** 10.3389/fnut.2025.1559027

**Published:** 2025-01-30

**Authors:** Asli Can Karaca, Giancarlo Cravotto, Hefei Zhao, Tawheed Amin, Luca Rastrelli

**Affiliations:** ^1^Department of Food Engineering, Istanbul Technical University, Maslak, Istanbul, Türkiye; ^2^Department of Drug Science and Technology, University of Turin, Turin, Italy; ^3^Department of Food Science and Technology, University of California, Davis, Davis, CA, United States; ^4^Division of Food Science and Technology, Sher-e-Kashmir University of Agricultural Sciences and Technology, Srinagar, India; ^5^Department of Pharmacy, University of Salerno, Fisciano, Salerno, Italy; ^6^National Biodiversity Future Center (NBFC), Palermo, Italy

**Keywords:** food processing technologies, sustainability, bioactive compounds, advanced analytical techniques, polyphenols, green revolution

The global food industry is currently facing increasing pressure to improve product quality while simultaneously addressing health concerns, sustainability, and resource efficiency (1). Advances in food processing technologies, quality analysis, and green practices are central to overcoming these challenges. The Research Topic *Advances in Food Processing and Analysis: Product Quality and Green Revolution* explores innovative approaches that integrate cutting-edge technologies, bioactive compounds, and sustainable food production practices. This Editorial highlights the key contributions of the articles in this Research Topic, positioning them within the broader movement toward a more sustainable and health-conscious food system.

## Recent advances in food extraction and processing

In recent decades, significant progress has been made, however, the shift toward a greener industrial revolution remains a long-term endeavor. Innovative heating technologies, such as microwaves, radio frequencies, infrared, and ohmic heating, have substantially enhanced food processing in terms of product quality and energy efficiency. Meanwhile, non-thermal processes such as hydrodynamic cavitation and pulsed electric fields (PEF) have emerged as viable alternatives to high-pressure processing (HPP) for the cold pasteurization of fruit juices. The valorization of by-products through the recovery of primary and secondary metabolites using highly efficient technologies and green solvents holds great potential for improving production sustainability (2, 3). Subcritical water extraction for medium- to high-polarity compounds and supercritical CO_2_ extraction for a polar molecules enable the replacement of organic solvents, contributing to more sustainable processing methods (4).

## Advanced analytical techniques for food quality control

One central theme of this Research Topic is the application of advanced analytical tools to improve food quality control. The contribution of using the electronic tongue and high-performance liquid chromatography (HPLC) to analyze *Ganoderma lucidum* is a prime example of how innovative technologies can rapidly assess food quality. Combining sensory properties with the quantification of bioactive triterpenes, such as ganoderic acids, Tian et al., offer an efficient method to assess the authenticity and quality of functional foods. This approach not only enhances product traceability but also reduces reliance on traditional, labor-intensive methods, thus improving both efficiency and consumer confidence.

Similarly, the study of Calatayud et al., on olive oil phenols illustrates the power of advanced chemical analysis, using techniques like UPLC-DAD, to monitor the oxidative stability and phenolic content of olive oil over time. This work underscores the importance of cultivar-specific responses to storage, revealing that certain olive varieties, such as Leccino and Moraiolo, exhibit greater inhibition to oxidation. These insights are crucial for enhancing the quality of olive oil during storage, contributing to longer shelf life and improved nutritional value. Together, these studies demonstrate the transformative potential of modern analytical tools for ensuring high-quality, safe, and sustainable food products (5).

## Bioactive natural compounds: polyphenols and health benefits

Polyphenols, renowned for their antioxidant, anti-inflammatory, and antibacterial properties, are another key area of focus in food quality and sustainability Sun et al.. The mini-review on polyphenols, presented by Sun et al., offers a comprehensive overview of their health benefits, particularly in the context of antibiotic resistance and the prevention of chronic diseases. As plant-derived bioactive compounds, polyphenols align with the growing demand for natural, functional ingredients in the food industry. Their role in food processing extends beyond health benefits, as optimizing polyphenol extraction methods can lead to greener and more sustainable processing practices. Furthermore, enhancing food safety, extending shelf life, and improving overall product quality through polyphenols without compromising environmental sustainability positions these compounds as pivotal in the green revolution of the food system. This review also emphasizes the importance of advanced analytical techniques, such as spectroscopy and chromatography, for accurately measuring polyphenol content and efficacy in food products.

## Innovations in food ingredient modification: improving stability and texture

Another area of significant innovation within food processing is the modification of food ingredients to improve texture, stability, and overall quality with the application of novel food processing technologies. The study of Ji et al., on atmospheric pressure plasma jet (APPJ) treatment of wheat starch highlights how plasma treatment can alter the physicochemical properties of starch, such as reducing viscosity and increasing solubility. The effects of APPJ treatment on wheat starch's rheological properties and surface morphology suggest that plasma treatment can be an effective strategy for creating modified ingredients with specific functional characteristics, catering to modern consumer demands for convenience and high-quality foods. Similarly, the experimental article on acetylated distarch phosphate (ADSP) in oyster sauce (Li et al.) emphasizes the potential of modified starches to enhance the texture, water retention, and long-term stability of food products. This research shows that starch derivatives can optimize the rheological properties of sauces, creating a smoother, more stable product that is less prone to separation. Such innovations not only improve the sensory quality of food but also contribute to sustainability by extending shelf life and reducing food waste.

## Fermentation and sustainable beverage production

Fermentation remains one of the most promising technologies for improving the sensory and nutritional properties of foods and beverages (6). The study on the fermentation of sea buckthorn juice provides valuable insights into the metabolic changes that occur during yeast-assisted fermentation. By employing techniques such as gas chromatography-mass spectrometry (GC-MS) and ultra-high-performance liquid chromatography-mass spectrometry (UHPLC-MS), Peng et al., identify significant increases in volatile aroma compounds and non-volatile metabolites, which improve the flavor and nutritional profile of the juice. The results highlight the impact of fermentation on enhancing the organoleptic qualities of sea buckthorn juice, making it more palatable for consumers, while simultaneously increasing the content of bioactive compounds like glutathione and vanillin. This research underscores the potential of fermentation not only to improve flavor but also to enhance the bioactive properties of functional beverages. These findings suggest that fermentation can be a key process in developing sustainable, health-promoting beverages with improved sensory profiles, offering new opportunities for the food and beverage industry.

## Conclusion

### Toward a sustainable and innovative future in food systems

The articles published in this Research Topic contribute significantly to the ongoing green revolution in food systems. By embracing innovative processing technologies, natural bioactive compounds, and sustainable ingredient sourcing, the food industry can address its most pressing challenges, including health concerns, environmental impact, and food waste. The integration of advanced analytical tools and sustainable food practices is essential for improving product quality and ensuring food safety, while simultaneously reducing the environmental footprint of food production. These studies collectively reflect the growing need for interdisciplinary approaches that combine food science, biotechnology, and environmental sustainability. As the food industry continues to evolve, it is clear that innovations in food processing, such as the use of functional ingredients such as polyphenols and the development of modified starches, will play a critical role in shaping the future of food production. The research featured in this Research Topic highlights the dynamic and rapidly evolving landscape of food processing, where health, sustainability, and innovation converge to create a more resilient and sustainable food system. We encourage further research in these areas to continue exploring novel approaches for improving food quality, ensuring food safety, and advancing sustainable practices across the food industry.
